# Temperature Effects on Expression Levels of *hsp* Genes in Eggs and Second-Stage Juveniles of *Meloidogyne hapla* Chitwood, 1949

**DOI:** 10.3390/ijms25094867

**Published:** 2024-04-29

**Authors:** Łukasz Flis, Tadeusz Malewski, Renata Dobosz

**Affiliations:** 1 Museum and Institute of Zoology, Polish Academy of Sciences, Twarda 51/55, 00-818 Warsaw, Poland; lflis@miiz.waw.pl; 2 Department of Entomology and Animal Pests, Institute of Plant Protection-National Research Institute, Węgorka 20, 60-318 Poznan, Poland; r.dobosz@iorpib.poznan.pl

**Keywords:** temperature, incubation time, expression, heat shock genes, *Meloidogyne hapla*, bioindicator

## Abstract

*Meloidogyne hapla* is one of the most important nematode pathogens. It is a sedentary, biotrophic parasite of plants that overwinters in the soil or in diseased roots. The development of *M. hapla* is temperature dependent. Numerous studies have been performed on the effect of temperature on the development of *M. hapla*, but only a few of them analyzed the heat shock protein (*hsp*) genes. The aim of the study was to perform expression profiling of eight *hsp* genes (*Mh-hsp*90, *Mh-hsp*1, *Mh-hsp*4, *Mh-hsp*6, *Mh-hsp*60, *Mh-dnj*19, *Mh-hsp*43, and *Mh-hsp*12.2) at two development stages of *M. hapla*, i.e., in eggs and second-stage juveniles (J2). The eggs and J2 were incubated under cold stress (5 °C), heat stress (35 °C, 40 °C), and non-stress (10 °C, 20 °C, and 30 °C) conditions. Expression profiling was performed by qPCR. It was demonstrated that only two genes, *Mh-hsp*60 and *Mh-dnj*19, have been upregulated by heat and cold stress at both development stages. Heat stress upregulated the expression of more *hsp* genes than cold stress did. The level of upregulation of most *hsp* genes was more marked in J2 than in eggs. The obtained results suggest that the *Mh-hsp*90 and *Mh-hsp*1 genes can be used as bioindicators of environmental impacts on nematodes of the *Meloidogyne* genus.

## 1. Introduction

*Meloidogyne hapla* Chitwood, 1949 (northern root-knot nematode) is one of the most important nematode pathogens. It is a sedentary, biotrophic parasite of plants and it overwinters in the soil or in diseased roots. *M. hapla* occurs in cold regions of crop production where the mean temperature is −15 °C in the coldest month and approximately 27 °C in the warmest month [1]. The amount of yield loss varies according to the host status, agronomic practices, and environmental conditions. *Meloidogyne* spp. are poikilothermic animals, which is why soil temperature impacts on the rate of development and hence the number of generations that the nematode will complete in a cropping cycle, as well as the rate of population growth and, consequently, the crop yield losses [2].

Temperature acclimation is an adaptive response of organisms to low or high temperature that increases their capacity to tolerate freezing or heating. Numerous studies indicate that heat shock protein (Hsp proteins) play an important role in the process of nematode adaptation to the environment [3,4,5]. To date, the heat shock genes (*hsp* genes) and the Hsp proteins they encode have been best studied and described in the free-living bacterivorous nematode *Caenorhabditis elegans* (Maupas, 1900). Studies have shown that the expression of *hsp* genes influences the lifespan of *C. elegans* [6,7,8]. It has also been shown that *hsp* genes in *C. elegans* are induced in response to various stressors, such as oxidative stress [9,10,11], electromagnetic field [12,13,14], immunological stress [15,16,17], or toxic chemical compounds [18,19,20]. However, thermal stress is one of the stress factors most often used in experiments aimed at activating *hsp* genes in *C. elegans* [21,22,23].

Research on *hsp* genes was also carried out on nematodes parasitizing plants belonging to the genus *Meloidogyne*. Studies of the *hsp* genes in this group of nematodes have so far focused mainly on the *hsp*90 gene and the Hsp90 protein encoded by this gene [24,25,26]. Based on the study results, it was also found that the *hsp*90 gene of *Meloidogyne artiellia* Franklin, 1961 is homologous to the *daf*-21 gene in *C. elegans* [24]. The influence of temperature on the expression of the *hsp*90 gene in *M. artiellia* and *M. incognita* was also studied [24,25]. Studies on the *Mh-hsp*90 gene as well as the *Mh-hsp*1, *Mh-hsp*60, *Mh-hsp*43, and *Mh-hsp*12.3 genes in *M. hapla* showed an increase in the expression of this gene as a result of the effect of the diffusate derived from *Vicia sativa* L. seeds [27]. The influence of stress-inducing temperatures and organic compounds on the expression of the *hsp*90 gene was also studied in *M. hapla* [28]. The genome of *M. hapla* is the smallest among the genomes of multicellular animals examined so far [29]. A sequenced genome opens the possibility of identifying *hsp* genes and studying their response to environmental stressors. The results of such research would complement the current knowledge about the life processes of *M. hapla* and, more broadly, nematodes of the *Meloidogyne* genus.

This article presents an original study of the effect of different temperatures and incubation times on the expression of selected *hsp* genes in eggs and second-stage juveniles (J2) of northern root-knot nematode.

## 2. Results

### 2.1. Identification of hsp Genes in the Meloidogyne hapla Genome

*Hsp* genes (Table 1) were identified by BLASTn search of genomic *M. hapla* (PRJNA29083–VW9) sequence against *C. elegans* (PRJNA13758) at WormBase ParaSite (version WS285) [30].

The applicable primers located on different exons of the *Mh-hsp*4, *Mh-hsp*6, *Mh-dnj*19, and *Mh-hsp*12.2 genes (Table S1) were designed based on the *hsp* gene sequences found.

### 2.2. Expression Profiling of hsp Genes

Depending on the incubation time used, temperatures of 30 °C, 35 °C, and 40 °C were lethal for J2. When exposed to 40 °C, all J2 had died after 336 h of incubation. Individuals exposed to 35 °C were dead after 1008 h of incubation, whereas those exposed to 30 °C were dead after 1344 h of incubation. The variants of the experiments presented above, in which J2 died, were not used for further research, i.e., J2 that died as a result of being incubated in the above temperature and time combinations were not included in the *hsp* gene expression studies.

The influence of stress (5 °C—cold stress, 35 °C and 40 °C—heat stress) and non-stress temperatures (normal conditions) (10 °C, 20 °C—control temperature, 30 °C) on the expression of eight *hsp* genes in eggs and J2 of *M. hapla* was investigated. Expression profiling of *hsp* genes showed the association of their expression level with the *M. hapla* developmental stage, temperature, and incubation time.

**Figure 1:**
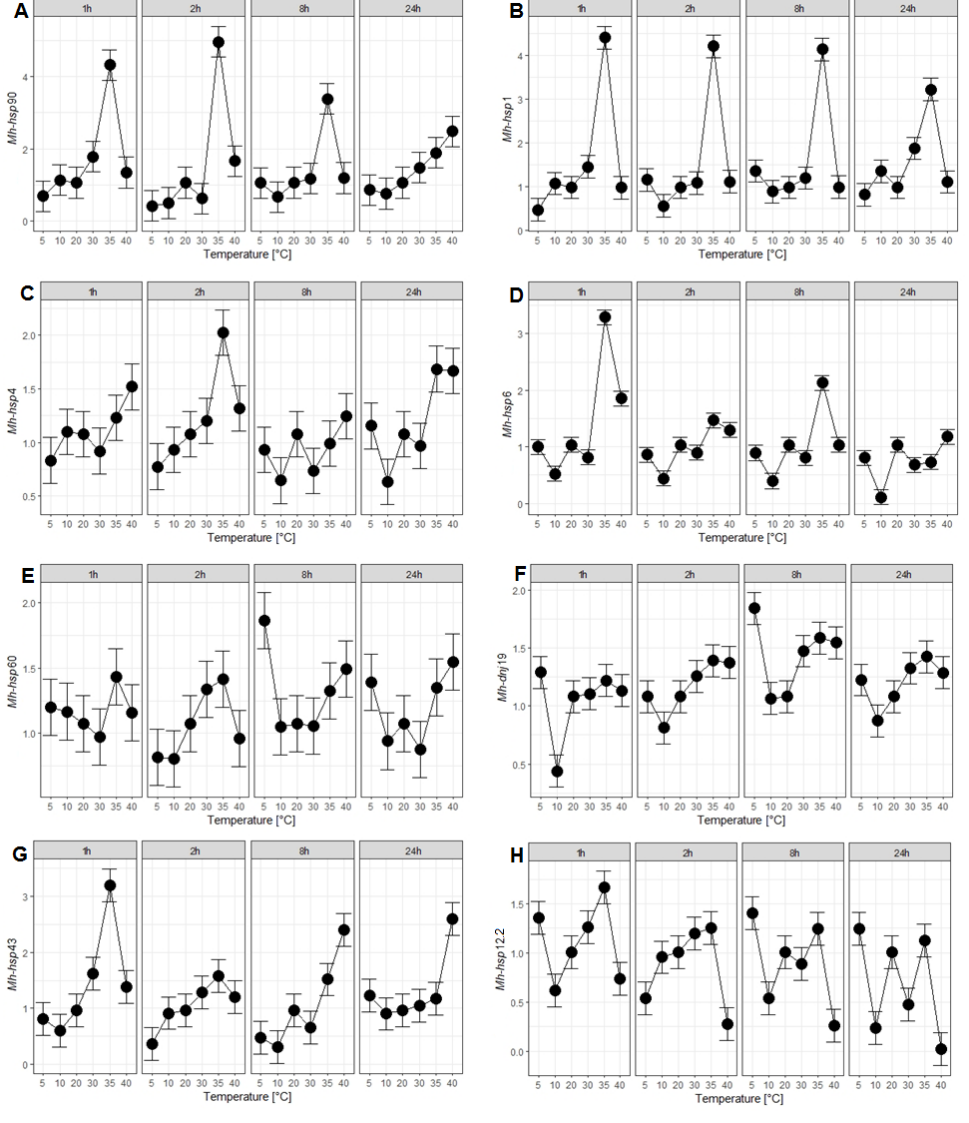
Expression profiling of *hsp* genes in the *Meloidogyne hapla* at the developmental stage of the egg: (**A**) *Mh-hsp*90, (**B**) *Mh-hsp*1, (**C**) *Mh-hsp*4, (**D**) *Mh-hsp*6, (**E**) *Mh-hsp*60, (**F**) *Mh-dnj*19, (**G**) *Mh-hsp*43, (**H**) *Mh-hsp*12.2 after 1, 2, 8 and 24 h of incubation time at the indicated temperature. Control temperature was 20 °C. Average effects (marginal means) estimated for the best model identified in the model selection are presented. The dot indicates the value of the estimate, the whiskers indicate the 95% confidence interval of this estimate.

**Figure 2:**
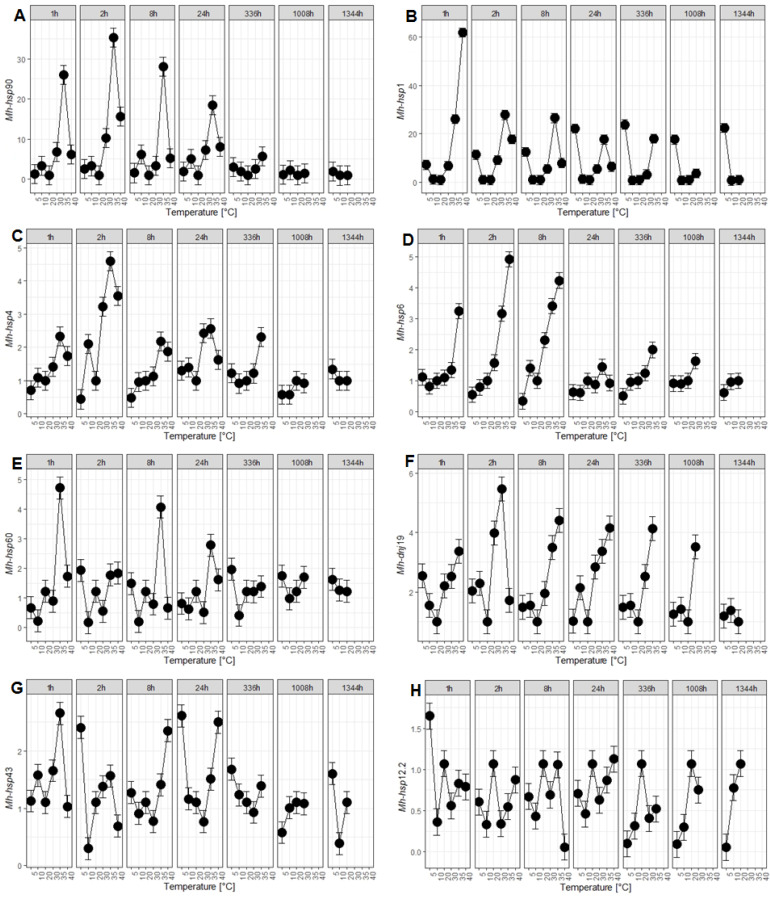
Expression profiling of *hsp* genes in the *Meloidogyne hapla* at second-stage juveniles: (**A**) *Mh-hsp*90, (**B**) *Mh-hsp*1, (**C**) *Mh-hsp*4, (**D**) *Mh-hsp*6, (**E**) *Mh-hsp*60, (**F**) *Mh-dnj*19, (**G**) *Mh-hsp*43, (**H**) *Mh-hsp*12.2 after 1, 2, 8 and 24 h of incubation time at the indicated temperature. Control temperature—20 °C. Average effects (marginal means) estimated for the best model identified in the model selection are presented. The dot indicates the value of the estimate, the whiskers indicate the 95% confidence interval of this estimate.

In both stages, an increase in the transcription level of all *hsp* genes was observed in response to heat stress, with the exception of the *Mh-hsp*12.2 gene in J2. Only two genes, *Mh-hsp*60 and *Mh-dnj*19, in both developmental stages upregulated their expression in response to heat and cold stress. As a result of cold stress, an increase in the expression of the *Mh-hsp*60, *Mh-dnj*19, and *Mh-hsp*12.2 genes was observed in both investigated developmental stages (Figure 1E,F,H and Figure 2E,F,H, respectively). The strongest response to heat and cold stress was observed in the *Mh-hsp*1 gene in J2 (Figure 2B). The second highest increase in the expression level as a result of heat stress was observed in the *Mh-hsp*90 gene in J2 (Figure 2A). It was also demonstrated that the *Mh-hsp*90, *Mh-hsp*4, and *Mh-hsp*6 genes did not respond to cold stress in both stages of *M. hapla* development (Figure 1A,C,D and Figure 2A,C,D). For the remaining genes, i.e., *Mh-hsp*1 and *Mh-hsp*43, an increase in expression caused by cold stress was found only in J2 (Figure 2B,G) (Table 2). In the conducted studies, differences in expression levels of *hsp* genes between the analysed developmental stages were also observed. In most of the analysed *hsp* genes (except for the *Mh-hsp*43 and *Mh-hsp*12.2 genes), higher expression levels were observed in J2 than in eggs.

### 2.3. Response of hsp Genes to Heat Stress

In eggs under heat stress, *Mh-hsp*1, *Mh-hsp*6, *Mh-hsp*43, and *Mh-hsp*12.2 genes were observed to respond after the shortest time of exposure (Figure 1B,D,G,H, respectively). The most effective temperature was 35 °C. Their expression reached the highest level after 1 h treatment and had increased from 1.7 (*Mh-hsp*12.2), 3.2 (*Mh-hsp*43), and 3.3 (*Mh-hsp*6) to 4.4 (*Mh-hsp*1) fold. Expression of *Mh-hsp*4 and *Mh-hsp*90 reached the highest level (2.0-fold and 4.96-fold increasing, respectively) after 2 h of heat stress treatment (Figure 1C,A, respectively). The expression of the *Mh-hsp*60 and *Mh-dnj*19 genes also increased, but to a lesser extent, reaching the maximum level after 24 h at 40 °C and after 8 h at 35 °C, respectively (Figure 2E,F).

Changes in the expression level of *hsp* genes in J2 were much more marked than in eggs. The highest (61.8-fold) and fastest (1 h of treatment) response to heat stress was found for *Mh-hsp*1 at 40 °C (Figure 2B). The response displayed by this gene to heat stress in J2 was over 14-fold stronger than was its response in eggs. This gene stood out from the other *hsp* genes in that its expression remained consistently high throughout the experiment. The fast (1 h) upregulation of gene expression by heat stress was also detected for *Mh-hsp*60 (Figure 2E) and *Mh-hsp*43 (Figure 2G) genes, but their expression levels were significantly lower (4.7-fold for *Mh-hsp*60 and 2.7-fold for *Mh-hp*43, correspondingly). Other genes, i.e., *Mh-hsp*90, *Mh-hsp*4, *Mh-hsp*6, and *Mh-dnj*19, required a longer (2 h) exposure to heat stress treatment to reach the maximum expression levels (35.3-fold for *Mh-hsp*90, 4.6-fold for *Mh-hsp*4, 4.9 fold for *Mh-hsp*6, and 5.5 fold for *Mh-dnj*19 (Figure 2A,C,D,F, respectively). *Mh-hsp*90 was also one of the genes whose expression increased the most in J2 (8-fold) compared to its expression level in eggs. The response of *Mh-hsp*43 and *Mh-hsp*60 to heat stress was biphasic. The highest expression level of *Mh-hsp*60 was detected after 1 h and 8 h of heat stress treatment (Figure 2E). *Mh-hsp*43 responded similarly to heat stress, but the expression of this gene after the long-term (8 and 24 h) treatment occurred not at 35 °C, but at 40 °C (Figure 2G).

### 2.4. Response of hsp Genes to Cold Stress

The effect of cold stress (5 °C) on the expression levels of *hsp* genes was more moderate than the effect of heat stress. In eggs, cold stress increased expression of only three (*Mh-hsp*60, *Mh-dnj*19, and *Mh-hsp*12.2; Figure 1E,F,H, respectively) from eight analyzed genes. Their expression increased from 1.4 fold for *Mh-hsp*12.2 and 1.8 fold for *Mh-dnj*19 to the highest level (1.9 fold) for the *Mh-hsp*60 gene.

*Mh-hsp*1, *Mh-hsp*60, *Mh-dnj*19, *Mh-hsp*43, and *Mh-hsp*12.2 genes responded to cold stress in J2. The highest level of expression among all investigated *hsp* genes was observed in the *Mh-hsp*1 gene (23.9 fold after 336 h incubation). The expression level of this gene remained consistently high throughout the entire experiment, just as it did in the case of heat stress (Figure 2B). Apart from the *Mh-hsp*1 gene, the only gene whose expression increase was observed after several incubation periods was the *Mh-hsp*43 gene. An increase in the expression of this gene was observed after 2 h (2.4 fold), 24 h (2.6 fold), 336 h (1.7 fold), and 1344 h (1.6 fold) (Figure 2G). In the *Mh-dnj*19 gene, a biphasic increase in expression was observed after 1 h (2.5 fold) and 2 h (2.0 fold) (Figure 2F). For the *Mh-hsp*60 (Figure 2E) and *Mh-hsp*12.2 (Figure 2H) genes, a single increase in expression was observed after 2 h (1.9 fold) and 1 h (1.7 fold) of incubation, respectively.

## 3. Discussion

As a result of the imposition of a stress factor (biotic or abiotic), metabolic changes occur in the cells. The cell’s response to stressful stimuli includes, among others, an increase in the expression of heat shock genes, and then the synthesis of heat shock proteins also called chaperones [31].

For *M. hapla*, only four *hsp* genes have been described [27]. In this work, we extended the annotation of the *M. hapla* genome and further identified four orthologs based on the *C. elegans hsp* gene sequences: *Mh-hsp*4, *Mh-hsp*6, *Mh-dnj*19, and *Mh-hsp*12.2 (Table 1).

According to the literature, the exposure to temperatures of 5 °C (cold stress) [32,33,34], 35 °C, and 40 °C (heat stress) [35,36,37] significantly inhibited the development of this nematode. In order to demonstrate the temperature effects on *hsp* gene expression, the individuals exposed to normal conditions (10 °C, 20 °C—control and 30 °C) [35,38] were also covered in the gene expression profiling exercise.

Among all analysed *hsp* genes, the highest increase in expression caused by heat and cold stress was observed in J2 in the *Mh-hsp*1 gene (Figure 2B), but only by heat stress in the *Mh-hsp*90 gene (Figure 2A). The Hsp90 and Hsp70 proteins encoded by these genes are the main chaperones in the cytosol of eukaryotic cells. They perform an important role in protein quality control by preventing the aggregation of proteins, catalysing the folding of newly synthesized proteins and promoting the degradation of denatured ones [39]. The very high increase in the expression of these genes observed in the study confirms their protective function in J2 stage of *M. hapla* against thermal stress. Both in the research presented in this paper and in that conducted by De Luca on *M. artiellia* [24], Bai on *M. incognita* [25], and Wu on *M. hapla* [28], it was established that the expression of the *hsp*90 gene was always significantly higher at temperatures causing heat stress in the studied developmental stages of *Meloidogyne*. A significant increase in the expression of this gene was observed not only during thermal stress but also during stress caused by heavy metals or inorganic compounds [24,25,27,28]. A clear increase in the expression of the *hsp*90 gene as a result of various environmental stressors suggests that this gene can be used as a potential bioindicator of the environmental impact on nematodes belonging to the genus *Meloidogyne* [25,28].

Among the examined *hsp* genes, the strongest expression upregulation is displayed by the *Mh-hsp1* gene in J2 under heat and cold stress (Figure 2B). The obtained results are in line with data obtained in studies of cyst forming nematodes of the genus *Globodera*, *G. rostochiensis* and *G. pallida*, where the influence of temperature on the expression level of this gene was demonstrated [40,41]. Heat stress upregulated *hsp*1 expression in these three species of nematodes. In *G. pallida*, the expression of *hsp*1 was upregulated not only by heat but also by cold stress [42]. Studies have shown that Hsp70 proteins perform a decisive role in acquiring thermotolerance, i.e., the cell’s resistance to high, often lethal, temperatures. The cells that are persistently resistant to thermal stress display consistently high levels of Hsp proteins [43]. Moreover, it was found that in *C. elegans* the number of *hsp*70A gene (*hsp*1 synonym) transcripts increases several times in response to heat shock and is primarily responsible for the increased thermotolerance of this species [44,45]. In *M. hapla*, the *Mh-hsp*1 gene is likely also responsible for acquiring thermotolerance in J2. Research on this gene in *C. elegans* has also proven its significant role in the early development of the juvenile stage and the regulation of the lifespan of this nematode [46,47,48]. It has also been shown that over expression of *hsp*1 leads to impaired motility of *C. elegans* [49].

Heat stress was found to increase the expression of the *Mh-hsp*4 (Figure 1C and Figure 2C) and *Mh-hsp*6 genes (Figure 1D and Figure 2D) in eggs and in J2 of *M. hapla*. The obtained results are consistent with those obtained on *C. elegans*, indicating that heat stress increases expression of *hsp*4 [50,51,52,53] and *hsp*6 genes [54,55,56]; however, the expression of these genes does not increase under cold stress [53].

The expression of the *Mh-hsp*60 gene in eggs and in J2 increased as a result of heat stress; however, under cold stress, it was the case only in eggs. (Figure 1E and Figure 2E). Experiments conducted on *C. elegans* and *Plectus acuminatus* regarding the increase in *hsp*60 gene expression as a result of heat stress confirm the results obtained in this study [57,58,59]. However, there are no comparative studies on the effect of cold stress on the expression of the *hsp*60 gene in *C. elegans* and other nematodes. The obtained results regarding the increase in the expression of the *Mh-hsp*60 gene in both developmental stages of *M. hapla* during cold stress necessitate additional experiments to more precisely examine the response of this gene to low stress temperatures.

The expression of the *Mh-dnj*19 gene displayed an increase in the expression of this gene in eggs and J2 of *M. hapla* exposed to heat and cold stress (Figure 1F and Figure 2F). The only research on the *dnj*19 gene was carried out on *C. elegans,* where an increase in the expression of this gene was observed during heat stress [60].

An increase in the expression of the *Mh-hsp*43 gene was demonstrated in *M. hapla* eggs in response to heat stress, whereas in J2 it was the case in response to heat and cold stress (Figure 1G and Figure 2G). Similar studies were conducted on *C. elegans*. Studies performed on this model organism showed an increase in *hsp*43 gene expression as a result of incubation at 37 °C for 2.5 h [61]. Studies on the nematode *Bursaphelenchus xylophilus* showed an increase in the expression of the *hsp*43 gene during a 4-h incubation at 30 °C compared to the expression of this gene examined in nematodes incubated at 20 °C [62]. The experiments described above confirm the research results obtained in this study regarding the increase in *Mh-hsp*43 gene expression during heat stress in both developmental stages of *M. hapla*.

Analysis of the *Mh-hsp*12.2 gene showed an increase in its expression as a result of heat stress and cold stress in eggs, whereas in J2 it was the case for cold stress only (Figure 1H and Figure 2H). The obtained results are consistent with those obtained in *C. elegans*, where an increase in the expression of this gene in eggs was demonstrated under the influence of heat stress [63]. However, Douglas did not observe an increase in the expression of this gene in mature *C. elegans* (wild strain N2) under heat stress [64]. Other studies have shown a slight increase in the expression of the *hsp*12.2 gene in the L3 stage of *C. elegans* under cold stress and no expression of this gene under heat stress [65].

The expression of *hsp* genes measured under the same conditions of temperature and incubation time differed between the examined developmental stages of *M. hapla*. In almost all *hsp* genes (except *Mh-hsp*43 and *Mh-hsp*12.2), higher expression levels were always observed in J2 as compared to the expression of these genes in eggs. This is consistent with results obtained on *M. artiella*. The expression of the *hsp*90 gene at 5 °C was higher in eggs compared to the expression of this gene in J2. However, the expression of this gene at 30 °C was higher in J2 than in eggs [24]. Studies on *M. incognita* showed that under normal conditions, the constitutive expression of the *hsp*90 gene was higher in eggs than in J2 [66]. Studies of the *daf*21 (*hsp*90) and *hsp*12.2 genes in *C. elegans* showed their diverse constitutive expression in each of the examined developmental stages of this nematode [65]. Perhaps the developmental stages examined in this study also show differences in the constitutive expression of *hsp* genes, while incubation of both stages at stress-inducing temperatures deepens these differences. Another reason for the lower expression of *hsp* genes in *M. hapla* eggs may be that it is less sensitive to abiotic environmental factors, including heat and cold stress. Vrain [67] showed that eggs and first-stage juveniles (J1) developing inside the egg are less sensitive to low temperatures than are J2. The eggshell, consisting of three layers, an outer vitelline, a middle chitin, and an inner lipid layer, probably forms an insulating protective barrier not only physically but also thermally [68]. Moreover, eggs are laid by the female into a gelatinous matrix, creating the egg mass. This gelatinous matrix holds the eggs together and protects them from extreme environmental conditions, including extreme temperatures [69].

The highest expression levels of the analysed *hsp* genes were most often observed after 1, 2, 8, and 24 h of incubation at a stress temperature. After that period, their expression decreased, especially in J2. This is in line with the results obtained for *C. elegans*, where changes in expression of the *dnj*12, *dnj*19, and *dnj*13 genes in 10-day old nematodes after heat stress were found to be much less marked than in 4-day nematodes [60]. In 1-day old *C. elegans*, the expression of the *hsp*70, *hsp*16.2, and *hsp*16.11 genes after heat stress was much higher than in 4- and 7-day old nematodes [70]. The reason for the decrease in expression levels of the studied genes may be attributable to damage to proteins associated with the aging of cells at the tested J2 individuals of *M. hapla*, as misfolding and the loss of functions of various proteins sensitive to temperature [70] were observed in *C. elegans* as a result of the aging of this organism.

The results of this study, as well as those of Bai [25] and Dobosz [27], suggest that *Mh-hsp*1 *and Mh-hsp*90 genes could be used as bioindicators to reflect the impact of the environmental impact more fully of root-knot nematodes. An interesting research aspect would also be to undertake research on the silencing of the expression of these two genes, *Mh-hsp*90 and *Mh-hsp*1, whose expression, as a result of heat stress, reached the highest levels in the presented research. This would provide scope and ample opportunity to learn the functions of these genes in *M. hapla*.

## 4. Materials and Methods

### 4.1. Meloidogyne hapla Culture

The individuals of *M. hapla* were harvested from the roots of carrots (*Daucus carota* L.) and morphologically and genetically identified in accordance with Karssen’s and Petersen and Vrain’s diagnostic protocols [71,72]. The nematodes were cultured on tomato plants (*Solanum lycopersicum* L., Moneymaker variety). Tomato plants were placed in phytotron chambers where they were grown in temperature conditions 20 °C/18 °C (day/night) and a 16/8 h photoperiod (day/night) until they developed from four to five full proper leaves. The seedlings were subsequently repotted to receptacles filled with soil (1:1 gravel and soil for the production of vegetables), which contained J2 of *M. hapla*, at a density of 50 individuals per 200 cm^3^ of the soil. After 60 days, the roots of the tomato plants were gently removed from the soil and rinsed with water. Egg masses (containing nematodes in the developmental stage of the egg) were mechanically obtained from the cleaned roots and placed in the water in Petri dishes (3.5 cm in diameter, 1 cm in height). Using a stereoscopic microscope (Leica M205 C), egg masses were segregated, and the stage of their development was assessed based on size and colour. Egg masses at an early stage of development are smaller, whitish, and contain eggs at the stage of embryonic development leading to the creation of the first-stage juveniles (J1). Mature egg masses are larger, brownish, and contain eggs at various stages of embryo development, as well as J1s and J2s ready to leave the eggs [24,73,74]. Only white egg masses were chosen for the egg stage tests. J2 individuals were obtained from all egg masses, both more or less mature. For this purpose, the egg masses were incubated in water in Petri dishes at the optimal temperature of 20 °C until J2 emerged from the eggshells [32].

### 4.2. Heat and Cold Stress

**Figure 3:**
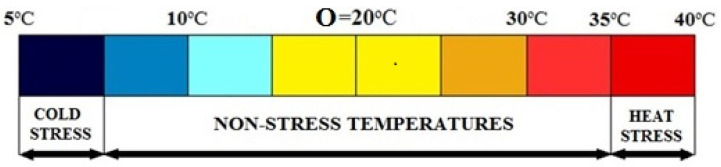
Incubation temperatures selected for the experiments (O–optimum temperature/control) (according to [32,33,36,67]).

The study was performed in three repetitions according to the modified methodology of De Luca [24]. Separately, one egg mass (egg stage) and 200 J2 (up to 24 h from hatching) were placed in a Petri dish each (3.5 cm in diameter, 1 cm in height) in distilled water (4 mL). Petri dishes, thus prepared, were incubated at the appropriate temperature for 1, 2, 8, and 24 h; J2 were additionally incubated for 336, 1008, and 1344 h at stress temperatures and in non-stress temperatures (normal conditions). The stress temperatures that significantly limited the development of northern root-knot nematode were found to be 5 °C, causing cold stress [32,33,34], and 35 °C and 40 °C, causing heat stress [35,36,37]. The temperature range from 10 °C to 30 °C constitutes normal conditions as the ontogenesis of this nematode species unfolds at these temperatures, progressing at the lowest rate at 10 °C and at the highest rate at 30 °C. [35,38]. The control temperature adopted throughout the duration of the experiment was 20 °C, the optimum temperature for development of *M. hapla* [36,75] (Figure 3).

The exposure of the nematodes at the mentioned temperatures was carried out in the incubator (Shaking incubator, NB-205V). After this exposure, the mortality of J2 was checked using a light microscope. Dead J2 were not used for tests. Then, both developmental stages of the nematodes were immediately transferred to Eppendorf tubes and preserved with phenosol (RNA preservative reagent by A&A Biotechnology RNA) and frozen at −80 °C, until the extraction of total RNA was obtained. RNA extraction, cDNA synthesis, primer design, and quantitative polymerase chain reaction (qPCR) were performed according to the methodology described in the publication by Dobosz [27].

### 4.3. Data Analyses

The values obtained for three independent tested samples were compared with the values calculated for control samples. For this purpose, the *t*-Student test was used [76]. The obtained data were modeled using generalized linear models (GLM) with a Gaussian (normal) error distribution [77]. All calculations were performed in the R computing environment [78]. The leading predictors in all models were temperature and incubation time, which were treated as categorical variables (factors). The reference level was 20 °C and the incubation time was 1 h. The model that was most suitable for the data was selected using model selection procedure, in which the fit of the models was assessed based on information theory criteria. In addition, packages from the “tidyverse” library were also used for calculations and visualization of results.

## 5. Conclusions

Thus far, *hsp* gene expression profiling has not been performed in *M. hapla*. It has been demonstrated for the first time that genes *Mh-hsp*90, *Mh-hsp*1, *Mh-hsp*4, *Mh-hsp*6, *Mh-hsp*60, *Mh-dnj*19, *Mh-hsp*43, and *Mh-hsp*12.2 respond to stress temperatures differently in eggs and in J2 of *M. hapla*. Changes in the expression level of most *hsp* genes (in addition to the *Mh-hsp*43 and *Mh-hsp*12.2 genes) were found to be more marked in J2 than in eggs. This suggests that eggs are less sensitive to stress temperatures. The obtained results also suggest that *Mh-hsp*90 and *Mh-hsp*1 genes can be used as bioindicators of the environmental impact on nematodes belonging to the genus *Meloidogyne*. On the other hand, the *Mh-hsp*1 gene may be involved in acquiring thermotolerance by J2 of *M. hapla*.

## Figures and Tables

**Table 1:** *Meloidogyne hapla* genes homologous to *Caenorhabditis elegans hsp* genes.

Hsp Family	Gene *C. elegans*	Gene *M. hapla*	*M. hapla* Gene Location on Contig
Hsp70	*hsp*4 (WBGene00002008)	*Mh-hsp*4 (MhA1_Contig349.frz3.gene2)	4430–7369
Hsp70	*hsp*6 (WBGene00002010)	*Mh-hsp*6 (MhA1_Contig349.frz3.gene2)	1610–3014
Hsp40	*dnj*19 (WBGene00001037)	*Mh-dnj*19 (MhA1_Contig579.frz3.gene6)	18539–19790
sHsps	*hsp*12.2 (WBGene00002011)	*Mh-hsp*12.2 (MhA1_Contig609.frz3.gene18)	43098–43583

**Table 2:** Increased expression level (+) or lack of response (−) of heat shock genes to heat and cold stress in eggs and in J2 of *Meloidogyne hapla*.

Heat Shock Protein Gene	Heat Stress		Cold Stress	
	Egg Stage	J2 Stage	Egg Stage	J2 Stage
*Mh-hsp*90	+	+	−	−
*Mh-hsp*1	+	+	−	+
*Mh-hsp*4	+	+	−	−
*Mh-hsp*6	+	+	−	−
*Mh-hsp*60	+	+	+	+
*Mh-dnj*19	+	+	+	+
*Mh-hsp*43	+	+	−	+
*Mh-hsp*12.2	+	−	+	+

## Data Availability

Data is contained within the article or Supplementary Materials.

## Supplementary Materials

The following supporting information can be downloaded at: https://www.mdpi.com/article/10.3390/ijms25094867/s1.

## References

[1] Wu X., Zhu X., Wang Y., Liu X., Chen L., Duan Y. (2018). The cold tolerance of the northern root-knot nematode, *Meloidogyne hapla*. PLoS ONE.

[2] Giné A., López-Gómez M., Vela M.D., Ornat C., Talavera M., Verdejo-Lucas S., Sorribas F.J. (2014). Thermal requirements and population dynamics of root-knot nematodes on cucumber and yield losses under protected cultivation. Plant Pathol..

[3] Zhao Y.L., Wang D.Y. (2012). Formation and regulation of adaptive response in nematode *Caenorhabditis elegans*. Oxid. Med. Cell. Longev..

[4] Weinstein D.J., Allen S.E., Lau M.C.Y., Erasmus M., Asalone K.C., Walters-Conte K., Deikus G., Sebra R., Borgonie G., Van Heerden E. (2019). The genome of a subterrestrial nematode reveals adaptations to heat. Nat. Commun..

[5] Fanelli E., Troccoli A., Tarasco E., De Luca F. (2021). Molecular characterization and functional analysis of the Hb-hsp90-1 gene in relation to temperature changes in *Heterorhabditis bacteriophora*. Front. Physiol..

[6] Manière X., Krisko A., Pellay F.X., Di Meglio J.M., Hersend P., Matic I. (2014). High transcript levels of heat-shock genes are associated with sorter lifespan of *Caenorhabditis elegans*. Exp. Gerontol..

[7] Richter K. (2015). daf-41/p23: A small protein heating up lifespan regulation. PLoS Genet..

[8] Jiang S., Jiang C.P., Cao P., Liu Y.H., Gao C.H., Yi-Xi X. (2022). Sonneradon a extends lifespan of *Caenorhabditis elegans* by modulating mitochondrial and IIS signaling pathways. Mar. Drugs.

[9] Li W.H., Shi Y.C., Chang C.H., Huang C.W., Liao V.H.C. (2014). Selenite protects *Caenorhabditis elegans* from oxidative stress via DAF-16 and TRXR-1. Mol. Nutr. Food Res..

[10] Chen W., Lin H.R., Wei C.M., Luo X.H., Sun M.L., Yang Z.Z., Chen X.Y., Wang H.B. (2018). Echinacoside, a phenylethanoid glycoside from *Cistanche deserticola*, extends lifespan of *Caenorhabditis elegans* and protects from Ab-induced toxicity. Biogerontology.

[11] Govindan S., Amirthalingam M., Duraisamy K., Govindhan T., Sundararaj N., Palanisamy S. (2018). Phytochemicals-induced hormesis protects *Caenorhabditis elegans* against α-synuclein protein aggregation and stress through modulating HSF-1 and SKN-1/Nrf2 signaling pathways. Biomed. Pharmacother..

[12] Cranfield C.G., Dawe A., Karloukovski V., Dunin–Borkowski R.E., de Pomerai D., Dobson J. (2004). Biogenic magnetite in the nematode *Caenorhabditis elegans*. Proc. R. Soc. Ser. B.

[13] Huang G.S., Yeh L.K., Chen Y.C. (2008). Nanoparticle-enhanced magnetic field induces apoptosis in nematodes. NSTI-Nanotech.

[14] Dawe A.S., Nylund R., Leszczynski D., Kuster N., Reader T., De Pomerai D.I. (2008). Continuous wave and simulated GSM exposure at 1.8 W/kg and 1.8 GHz do not induce hsp16-1 heat-shock gene expression in *Caenorhabditis elegans*. Bioelectromagnetics.

[15] Bolz D.D., Tenor J.L., Aballay A. (2010). A conserved PMK-1/p38 MAPK is required in *Caenorhabditis elegans* tissue-specific immune response to *Yersinia pestis* infection. JBC.

[16] Prithika U., Deepa V., Balamurugan K. (2016). External induction of heat shock stimulates the immune response and longevity of *Caenorhabditis elegans* towards pathogen exposure. Innate Immun..

[17] Eckl J., Sima S., Marcus K., Lindemann C., Richter K. (2017). Hsp90-downregulation influences the heat-shock response, innate immune response and onset of oocyte development in nematodes. PLoS ONE.

[18] Avila D.S., Benedetto A., Au C., Bornhorst J., Aschner M. (2016). Involvement of heat shock proteins on Mn-induced toxicity in *Caenorhabditis elegans*. BMC Pharmacol. Toxicol..

[19] Ezemaduka A.N., Wang Y., Li X. (2017). Expression of CeHSP17 protein in response to heat shock and heavy metal ions. J. Nematol..

[20] Li Y., Guan S., Liu C., Chen X., Zhu Y., Xie Y., Wang J., Ji X., Li L., Li Z. (2018). Neuroprotective effects of *Coptis chinensis* Franch polysaccharide on amyloid-beta (Aβ)-induced toxicity in a transgenic *Caenorhabditis elegans* model of Alzheimer’s disease (AD). Int. J. Biol. Macromol..

[21] Huang Y., Sterken M.G., Van Zwet K., Van Sluijs L., Pijlman G.P., Kamanga J.E. (2021). Heat stress reduces the susceptibility of *Caenorhabditis elegans* to orsay virus infection. Genes.

[22] Pagliuso D.C., Bodas D.M., Pasquinelli A.E. (2021). Recovery from heat shock requires themicroRNA pathway in *Caenorhabditis elegans*. PLoS Genet..

[23] Zhang Q.Y., Ji Y.T., Wang P., Xie H., Kong H.H., Qu C.Q. (2022). Paeoniflorin protects *Caenorhabditis elegans* from heat stress. Cur. Top. Nutraceutical Res..

[24] De Luca F., Di Vito M., Fanelli E., Reyes A., Greco N., De Giorgi C. (2009). Characterization of the heat shock protein 90 gene in the plant parasitic nematode *Meloidogyne artiellia* and its expression as related to different developmental stages and temperature. Gene.

[25] Bai C., Duan Y., Chen L., Liu Y., Zheng Y., Wang Y., Zhu X. (2014). Gene cloning and gene expression of Hsp90 from *Meloidogyne incognita* under the temperature and heavy metal stress. Int. J. Agric. Biol..

[26] Kundu A., Dutta A., Mandal A., Negi L., Malik M., Puramchatwad R., Antil J., Singh A., Rao U., Saha S. (2021). A comprehensive in vitro and in silico analysis of nematicidal action of essential oils. Front. Plant Sci..

[27] Dobosz R., Flis Ł., Bocianowski J., Malewski T. (2023). Effect of *Vicia sativa* L. on motility, mortality and expression levels of *hsp* genes in J2 stage of *Meloidogyne hapla*. J. Nematol..

[28] Wu X., Yu H., Yang R., Zhou Y., Zhu X., Wang Y., Liu X., Fan H., Chen L., Duan Y. (2019). Evaluation of suitable reference genes for gene expression analysis in the northern root-knot nematode; *Meloidogyne hapla*. PLoS ONE.

[29] Opperman C.H., Bird D.M., Williamson V.M., Rokhsar D.S., Burke M., Cohn J., Cromer J., Diener S., Gajan J., Graham S. (2008). Sequence and genetic map of *Meloidogyne hapla*: A compact nematode genome for plant parasitism. Proc. Natl. Acad. Sci. USA.

[30] WormBase ParaSite (version WS285). https://parasite.wormbase.org/index.html.

[31] Feder M.E., Hofmann G.E. (1999). Heat-shock proteins, molecular chaperones, and the stress response: Evolutionary and ecological physiology. Annu. Rev. Physiol..

[32] Vrain T.C., Barker K.R. (1978). Influence of low temperature on development of *Meloidogyne incognita* and *Meloidogyne hapla* eggs in egg masses. J. Nematol..

[33] Charchar J.M., Santo G.S. (2001). Effect of temperature on the embryogenic development and hatching of *Meloidogyne chitwoodi* races 1 and 2 and *M. hapla*. Nematol. Bras..

[34] Gulyas L., Powell J.R. (2022). Cold shock induces a terminal investment reproductive response in *C. elegans*. Sci. Rep..

[35] Thomason I.J., Lear B. (1961). Rate of reproduction of *Meloidogyne* spp. as influenced by soil temperature. Phytopathology.

[36] Kinloch R.A., Allen M.W. (1972). Interaction of *Meloidogyne hapla* and *M. javanica* infecting tomato. J. Nematol..

[37] Treinin M., Shliar J., Jiang H., Powell-Coffman J.A., Bromberg Z., Horowitz M. (2003). HIF-1 is required for heat acclimation in the nematode *Caenorhabditis elegans*. Physiol. Genom..

[38] Inserra R.N., Griffin G.D., Sisson D.V. (1983). Effects of temperature and root leachates on embryogenic development and hatching of *Meloidogyne chitwoodi* and *M. hapla*. J. Nematol..

[39] Hartl F.U., Bracher A., Hayer-Hartl M. (2011). Molecular chaperones in protein folding and proteostasis. Nature.

[40] den Akker S.E.-V., Laetsch D.R., Thorpe P., Lilley J.C., Danchin E.G.J., Da Rocha M., Rancurel C., Holroyd N.E., Cotton J.A., Szitenberg A. (2016). The genome of the yellow potato cyst nematode, *Globodera rostochiensis*, reveals insights into the basis of parasitism and virulence. Genome Biol..

[41] Jones L.M., Eves-Van den Akker S., Van-Oosten Hawle P., Atkinson H.J., Urwin P.E. (2018). Duplication of hsp-110 is implicated in differential success of *Globodera species* under climate change. Mol. Biol. Evol..

[42] Cymerys J., Niemiałtowski M. (2004). Heat shock proteins—A molecular perpetual motion machine. Postępy Biol. Komórki.

[43] Zhang H., Zhou Q., Yang Y., Chen X., Yan B., Du A. (2013). Characterization of heat shock protein 70 gene from *Haemonchus contortus* and its expression and promoter analysis in *Caenorhabditis elegans*. Parasitology.

[44] Snutch T.P., Heschl M.F.P., Baillie D.L. (1988). The *Caenorhabditis elegans* hsp70 genefamily: A molecular genetic characterization. Gene.

[45] Morley J.F., Morimoto R.I. (2004). Regulation of longevity in *Caenorhabditis elegans* by heat shock factor and molecular chaperones. Mol. Biol. Cell.

[46] Craig E.A. (2018). Hsp70 at the membrane: Driving protein translocation. BMC Biol..

[47] Papsdorf K., Sima S., Schmauder L., Peter S., Renner L., Hoffelner P., Richter K. (2019). Head-bent resistant Hsc70 variants show reduced Hsp40 affinity and altered protein folding activity. Sci. Rep..

[48] Papsdorf K., Sacherl J., Richter K. (2014). The balanced regulation of Hsc70 by DNJ-13 and UNC-23 is required for muscle functionality. J. Biol. Chem..

[49] Shen X., Ellis R.E., Lee K., Liu C.Y., Yang K., Solomon A., Yoshida H., Morimoto R., Kurnit D.M., Mori K. (2001). Complementary signaling pathways regulate the unfolded protein response and are required for *C. elegans* development. Cell.

[50] Yoneda T., Benedetti C., Urano F., Clark S.G., Harding H.P., Ron D. (2004). Compartment-specific perturbation of protein handling activates genes encoding mitochondrial chaperones. J. Cell Sci..

[51] Rodrigues A.J., Neves-Carvalho A., Teixeira-Castro A., Rokka A., Corthals G., Logarinho E., Maciel P. (2011). Absence of ataxin-3 leads to enhanced stress response in *C. elegans*. PLoS ONE.

[52] Dues D.J., Andrews E.K., Schaar C.E., Bergsma A.L., Senchuk M.M., Van Raamsdonk J.M. (2016). Aging causes decreased resistance to multiple stresses and a failure to activate specific stress response pathways. Aging.

[53] Labbadia J., Morimoto R.I. (2015). Repression of the heat shock response is a programmed event at the onset of reproduction. Mol. Cell.

[54] Labbadia J., Brielmann R.M., Neto M.F., Lin Y.F., Haynes C.M., Morimoto R.I. (2017). Mitochondrial stress restores the heat shock response and prevents proteostasis collapse during aging. Cell Rep..

[55] Kammenga J.E., Arts M.S.J., Oude-Breuil W.J.M. (1998). HSP60 as a potential biomarker of toxic stress in the nematode *Plectus acuminatus*. Arch. Environ. Contam. Toxicol..

[56] Lu M., Tan L., Zhou X.G., Yang Z.L., Zhu Q., Chen J.N., Luo H.R., Wu G.S. (2020). Tectochrysin increases stress resistance and extends the lifespan of *Caenorhabditis elegans* via FOXO/DAF-16. Biogerontology.

[57] Shi L., Yu X.T., Li H., Wu G.S., Luo H.R. (2023). D-chiro-inositol increases antioxidant capacity and longevity of *Caenorhabditis elegans* via activating Nrf-2/SKN-1 and FOXO/DAF-16. Exp. Gerontol..

[58] Kirstein J., Arnsburg K., Scior A., Szlachcic A., Guilbride D.L., Morimoto R.I., Bukau B., Nillegoda N.B. (2017). In vivo properties of the disaggregase function of J-proteins and Hsc70 in *Caenorhabditis elegans* stress and aging. Aging Cell.

[59] Fu R., Huang Z., Li H., Zhu Y., Zhang H. (2020). A hemidesmosome-to-cytoplasm translocation of small Heat Shock Proteins provides immediate protection against heat stress. Cell Rep..

[60] Wang F., Li D., Chen Q., Ma L. (2016). Genome-wide survey and characterization of the small heat shock protein gene family in *Bursaphelenchus xylophilus*. Gene.

[61] Sánchez-Blanco A., Rodríguez-Matellán A.G., Reis-Sobreiro M., Sáenz-Narciso B., Cabello J., Mohler W.A., Mollinedo F. (2014). *Caenorhabditis elegans* as a platform to study the mechanism of action of synthetic antitumor lipids. Cell Cycle.

[62] Douglas P.M., Baird N.A., Simic M.S., Uhlein S., McCormick M.A., Wolff S.C., Kennedy B.K., Dillin A. (2015). Heterotypic signals from neural HSF-1 separate thermotolerance from longevity. Cell Rep..

[63] Krause M. (2013). Structural and Functional Characterization of Small Heat Shock Proteins of the Nematode *Caenorhabditis elegans*. Ph.D. Thesis.

[64] Lourenço-Tessutti I.T., Souza Junior J.D., Martins-de-Sa D., Viana A.A.B., Carneiro R.M.D.G., Togawa R.C., de Almeida-Engler J., Batista J.A.N., Silva M.C.M., Fragoso R.R. (2015). Knock-down of heat-shock protein 90 and isocitrate lyase gene expression reduced root-knot nematode reproduction. Phytopathology.

[65] Vrain T.C., Barker K.R., Holtzman I.G. (1978). Influence of low temperature on rate of development of *Meloidogyne incognita* and *Meloidogyne hapla* larvae. J. Nematol..

[66] Curtis R.H., Robinson A.F., Perry R.N., Perry R.N., Moens M., Starr J.L. (2009). Hatch and host location. Root-Knot Nematodes.

[67] Sharon E., Spiegel Y. (1993). Glycoprotein characterization of the gelatinous matrix in the root-knot nematode *Meloidogyne javanica*. J. Nematol..

[68] Ben-Zvi A., Miller E.A., Morimoto R.I. (2009). Collapse of proteostasis represents an early molecular event in *Caenorhabditis elegans* aging. Proc. Natl. Acad. Sci. USA.

[69] Karssen G. (2002). The Plant Parasitic Nematode Genus Meloidogyne Göldi, 1892 (Tylenchida) in Europe.

[70] Petersen D.J., Vrain T.C. (1996). Rapid identification of *Meloidogyne chitwoodi*, *M. hapla* and *M. fallax* using PCR primers to amplify their ribosomal intergenic spacer. Fundam. Appl. Nematol..

[71] Evans A.A.F., Perry R.N., Perry R.N., Moens M., Starr J.L. (2009). Survival mechanisms. Root-Knot Nematodes.

[72] Díaz-Manzano F.E., Olmo R., Cabrera J., Barcala M., Escobar C., Fenoll C. (2016). Long-term in vitro system for maintenance and amplification of root-knot nematodes in *Cucumis sativus* roots. Front. Plant Sci..

[73] Bird A.F., Wallace H.R. (1965). The influence of temperature on *Meloidogyne hapla* and *Meloidogyne javanica*. Nematologica.

[74] Tournayre J., Reichstadt M., Parry L., Fafournoux P., Jousse C. (2019). “Do my qPCR calculation”; a web tool. Bioinformation.

[75] Biecek P. (2013). Data Analysis with R. Linear Models with Fixed, Random and Mixed Effects.

[76] R Core Team (2022). R: A languague and Environment for Statistical Computing.

[77] Burnham K.P., Anderson D.R. (2002). Model Selection and Multimodel Inference: A Practical Information-Theoretic Approach.

[78] Wickham H., Averick M., Bryan J., Chang W., D’Agostino McGowan L., Romain F., Grolemund G., Hayes A., Henry L., Hester J. (2019). Welcome to the tidyverse. JOSS.

