# Supplementary material for: Temperature Effects on Expression Levels of hsp Genes in Eggs and Second-Stage Juveniles of Meloidogyne hapla Chitwood, 1949
Source: Int J Mol Sci. 2024 Apr 29;25(9):4867. doi: 10.3390/ijms25094867 (PMC11084963; doi:10.3390/ijms25094867)
Supplement: Supplementary file 1 [file ijms-25-04867-s001.zip › ijms-2905385-supplementary.pdf]

Table S1. List of primers used in the study (F–Forward primer, R–Reverse primer).

Gen	Position in gene	Primer sequence (5′-3′)	Amplicon size (bp)	Annealing temperature (°C)	Author of primer
*Mh-hsp*90	1391-1412<br>1553-1572	F: TCTCTGATGATGAGGCTGAAGA<br>R: TCACCGTCCTTCTTGTCCTT	130	60.1<br>59.7	[27]
*Mh-hsp*1	1410-1431<br>1671-1694	F: ACTCATCTTGGTGGTGAAGATT<br>R: TCAATGCCATCAAAGAGAGAATCA	200	57.4<br>57.4	[27]
*Mh-hsp*4	1866-1885<br>2046-2065	F: GAAGGAGAACGCCCAATGAC<br>R: CCAGTTCCTTTGTCTTCGGC	158	58.9<br>58.9	this article
*Mh-hsp*6	15-34<br>310-332	F: TCGTCCATCTTTCAACCGTT<br>R: AACAGATTGTCTAATTGCTGGAG	218	58.1<br>58.2	this article
*Mh-hsp*60	909-929<br>1250-1269	F: TTCCTGCTCTTGAATTGGCT<br>R: AATTGTGACTTCATCCGCCT	218	58.1<br>58.2	[27]
*Mh-dnj*19	983-1002<br>1191-1210	F: TGTGAACATTGCAGTGGTGG<br>R: TCTCCAGGCTCAATTCCAGG	185	58.0<br>59.1	this article
*Mh-hsp*43	4156-4178<br>4407-4425	F: CGTAGAGAAGAATTCCGTGAAGA<br>R: TTCAGAGCGGTGACTTCCA	138	58.7<br>59.2	[27]
*Mh-hsp*12.2	709-728<br>888-907	F: GCCCCTTCAACACACAGATG<br>R: TACCCTTTCGTGGCTCATGA	156	59.1<br>58.7	this article
